# Blue and white light emission from zinc oxide nanoforests

**DOI:** 10.3762/bjnano.6.255

**Published:** 2015-12-23

**Authors:** Nafisa Noor, Luca Lucera, Thomas Capuano, Venkata Manthina, Alexander G Agrios, Helena Silva, Ali Gokirmak

**Affiliations:** 1 Department of Electrical and Computer Engineering, University of Connecticut, Storrs, Connecticut 06269, USA; 2 ZAE Bayern - Solar Factory of the Future, Fürtherstrasse 250, 90429 Nürnberg, Germany; 3 National Instruments, Austin, Texas 78759, USA; 4 Fraunhofer Center for Energy Innovation (CEI), University of Connecticut, Storrs, Connecticut 06269, USA; 5 Department of Civil and Environmental Engineering, University of Connecticut, Storrs, Connecticut 06269, USA,; 6 Center for Clean Energy Engineering, University of Connecticut, Storrs, Connecticut 06269, USA

**Keywords:** electrical stress, light emission, nanoforest, plasma, zinc oxide

## Abstract

Blue and white light emission is observed when high voltage stress is applied using micrometer-separated tungsten probes across a nanoforest formed of ZnO nanorods. The optical spectrum of the emitted light consistently shows three fine peaks with very high amplitude in the 465–485 nm (blue) range, corresponding to atomic transitions of zinc. Additional peaks with smaller amplitudes in the 330–650 nm range and broad spectrum white light is observed depending on the excitation conditions. The spatial and spectral distribution of the emitted light, with pink–orange regions identifying percolation paths in some cases and high intensity blue and white light with center to edge variations in others, indicate that multiple mechanisms lead to light emission. Under certain conditions, the tungsten probe tips used to make electrical contact with the ZnO structures melt during the excitation, indicating that the local temperature can exceed 3422 °C, which is the melting temperature of tungsten. The distinct and narrow peaks in the optical spectra and the abrupt increase in current at high electric fields suggest that a plasma is formed by application of the electrical bias, giving rise to light emission via atomic transitions in gaseous zinc and oxygen. The broad spectrum, white light emission is possibly due to the free electron transitions in the plasma and blackbody radiation from molten silicon. The white light may also arise from the recombination through multiple defect levels in ZnO or due to the optical excitation from solid ZnO. The electrical measurements performed at different ambient pressures result in light emission with distinguishable differences in the emission properties and *I*–*V* curves, which also indicate that the dielectric breakdown of ZnO, sublimation, and plasma formation processes are the underlying mechanisms.

## Introduction

The interest in environmentally friendly semiconductors, biocompatible [1], functional nanostructures [2], nanoscale electronic devices and large area electronics has led to significant research efforts in metal-oxide semiconductors such as ZnO. ZnO is a common, low-cost, antibacterial [3] material that forms various nanostructures depending on the process conditions. It is a direct and wide band gap semiconductor (≈3.4 eV) with large exciton binding energy (60 meV). ZnO nanowires have been shown to yield stimulated emission with optical pumping (e.g., nanowire laser) [4] and have been demonstrated as photodetectors [5]. ZnO films have also been used in transparent thin film transistors [6] and as phosphors [7]. ZnO typically grows as an n-type semiconductor due to oxygen deficiencies and has been used as the n-type material in heterojunctions [8], but there have also been significant efforts to produce high-quality p-type ZnO to form homojunction light emitting diodes (LED) that can produce UV [9], blue [10] or white light [11]. Recently there have also been reports on dye-sensitized solar cells [12–13] that utilize ZnO nanostructures. ZnO, with its interesting electronic and optical properties [14] and possibility of synthesis using relatively simple approaches, can become a low-cost alternative to GaN [8]. It is also one of the substances that sublimate congruently at atmospheric pressure [15–17]:

[1]
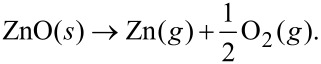


The results we present here are from electrical experiments performed on ZnO nanoforests formed by high-density nanowires grown on highly doped silicon microstructures. We have observed light emission upon application of relatively high electric fields (≈3–7 V/µm) to the nanoforests using tungsten probes. We have performed electrical characterization at different ambient pressures and analyzed the resulting optical emission spectra and videos to determine the mechanisms giving rise to light emission.

## Experimental

### ZnO growth

**Figure 1:**
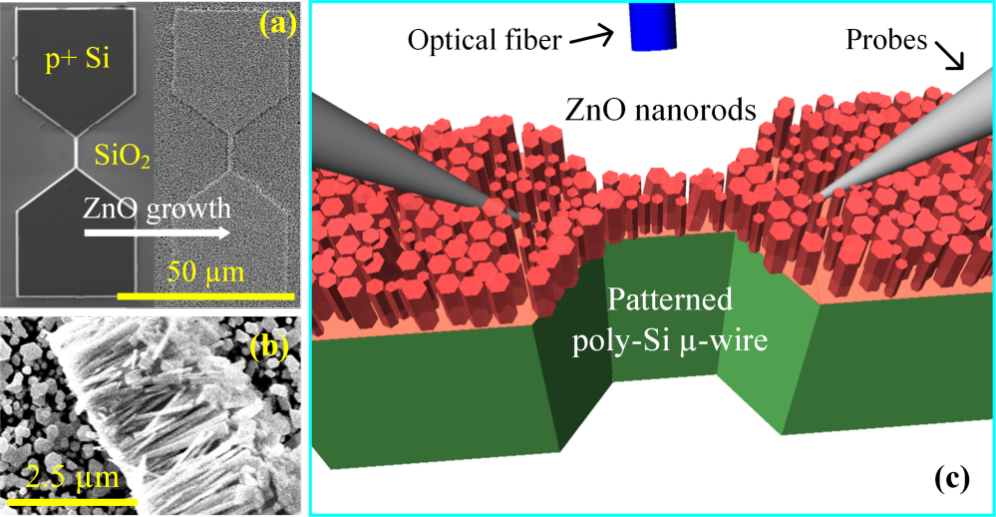
(a) SEM image of the ZnO nanorods grown on ≈100 nm thick, highly doped, patterned, p-type, silicon microstructures (before and after ZnO nanorod growth), (b) cross-section SEM image of the ZnO nanorods, and (c) schematic of the electrical probe and optical fiber arrangement.

Chemical bath deposition (CBD), a low-cost solution-based technique [18–19], is used to grow ZnO nanorods on oxidized silicon wafers with previously fabricated, highly doped, p-type, nanocrystalline silicon microstructures. The samples were precleaned by sonication in ethanol, dried with nitrogen and spin-coated with a seed solution that was prepared by dissolving 0.0457 mg of zinc acetate dihydrate in 50 mL of ethanol. The samples were then baked at 350 °C for 30 min and then submersed in a water-based precursor solution containing 25 mM of zinc nitrate hexahydrate, 25 mM of hexamethylenetetramine and 6 mM of poly(ethyleneimine) for 24 h, and kept at 90 °C. The process yields 2–2.5 μm long ZnO nanorods homogeneously grown along the *c*-direction of the wurtzite structure with 50–250 nm diameter (Figure 1) on top of a very thin layer of ZnO film (≈2–3 nm).

### Experimental setup

DC voltage sweeps between 0–80 V, AC sinusoidal voltages with peak-to-peak amplitudes up to 80 V, and 1–10 kHz frequency and microsecond single pulses with amplitudes up to 50 V were applied to the samples in open air and in vacuum in a probe station, which was equipped with high-magnification optics and high-resolution micromanipulators. The DC, AC, and pulse excitations were applied using a HP 4145B semiconductor parameter analyzer, a Tektronix AFG 3102 arbitrary/function generator, and an Agilent 8114A pulse generator, respectively. The atmospheric pressure measurements were performed in an Alessi probe station and those in vacuum were done in a Janis cryogenic probe station at 2.5 mTorr (medium vacuum).

The tungsten probe tips with 2.4 µm tip radius and 45° angle (Cascade Microtech, PTT-24/4-25) were forced to slightly slide through the ZnO nanoforest in order to ensure good electrical contact (Figure 1c). The probe separation for all electrical measurements was ≈10–15 µm. Either a high-sensitivity 1080p HD camcorder with a frame rate of 60 fps (Sony, HDR-CX160) or a high-speed camera with a maximum of 1200 fps (Casio, EX-F1) was connected to the microscope head for imaging during the measurements. In order to understand the physical mechanisms that lead to light emission, a wideband (200–1100 nm) optical spectrometer with 1 nm resolution (Ocean Optics, HR2000+) coupled with an optical fiber was incorporated with the system for spectral analysis. The fiber tip was attached to a probe arm and was aligned to the test area using a micromanipulator.

A high-speed PIN diode with built-in amplifier was attached to another micromanipulator and was positioned to face the test area in order to detect the emitted light intensity with better time resolution. The distance from the PIN diode to the sample was adjusted to achieve adequate signal-to-noise ratio without saturating the diode during the experiments. The voltage output of the built-in amplifier was significantly larger than any noise or perturbations coupled into the signal lines caused by the fast pulses. Hence, this approach was preferred over a simple PIN diode for detection. The applied voltage, PIN diode output voltage, and resulting current were measured simultaneously using two synchronized two-channel oscilloscopes (Tektronix, TDS 2002B) for the AC and pulse measurements.

**Figure 2:**
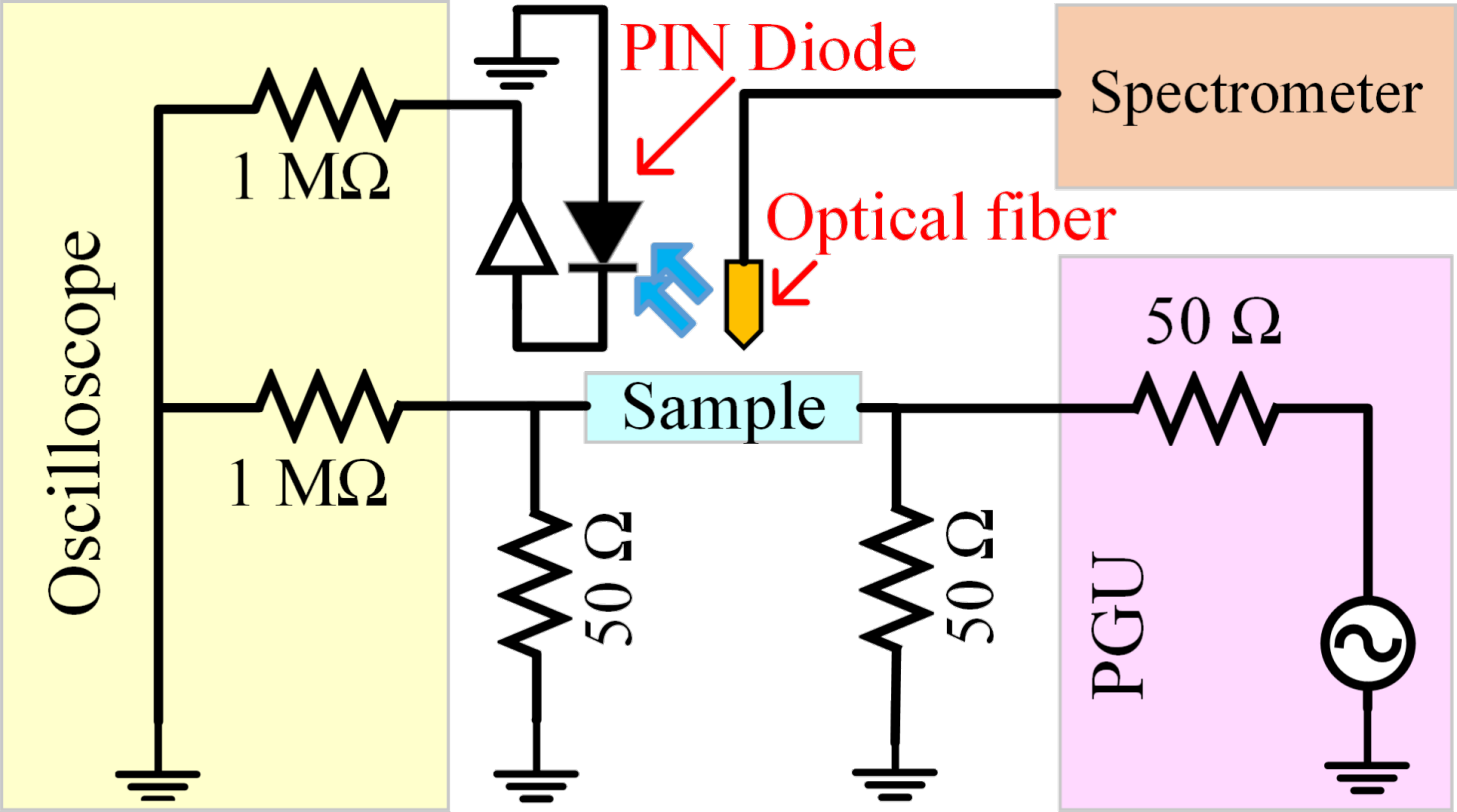
The schematics of a typical measurement setup with a pulse generating unit (PGU). An optical spectrometer was used to perform spectral analysis with time resolution of ≥1 ms in the 200–1100 nm range.

The coaxial cable lengths were matched to minimize the difference in phase delay. The output of the signal generator and the inputs to the oscilloscopes were terminated to 50 Ω resistors to eliminate reflections and oscillations (Figure 2). A black enclosure surrounding the whole setup was used and the other room light sources were turned off during the measurements to eliminate ambient light. The measurements were controlled through a computer.

The electrical resistivities of the bare polysilicon substrate and the sample with ZnO nanorods on polysilicon were measured using a four-point probe method.

## Results and Discussion

**Figure 3:**
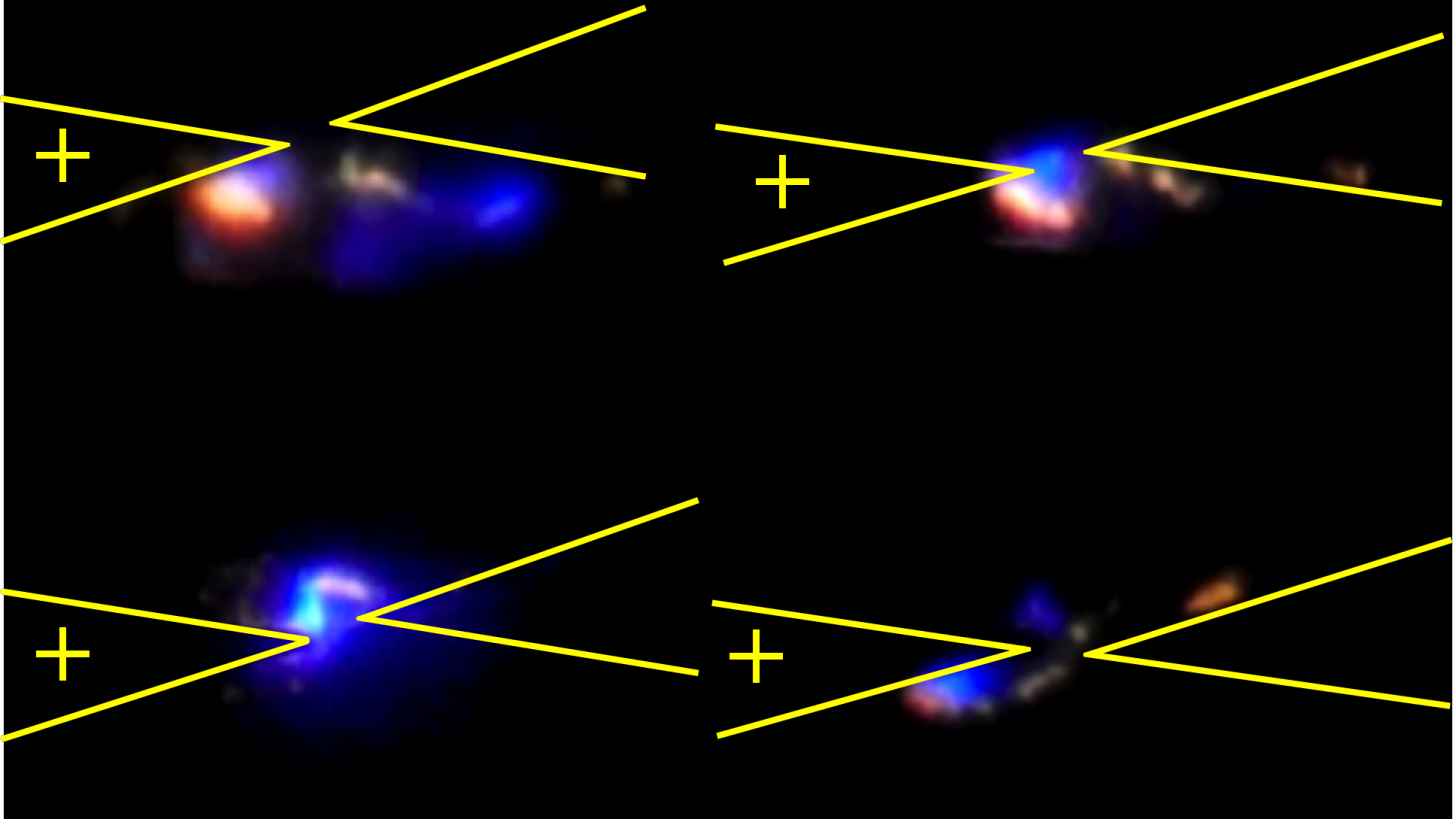
Frames extracted from high-speed videos showing light emission and changing percolation paths during DC tests with an approximate probe distance of 10–15 μm. The probe locations and applied voltage polarity are as indicated in yellow (multimedia view).

**Figure 4:**
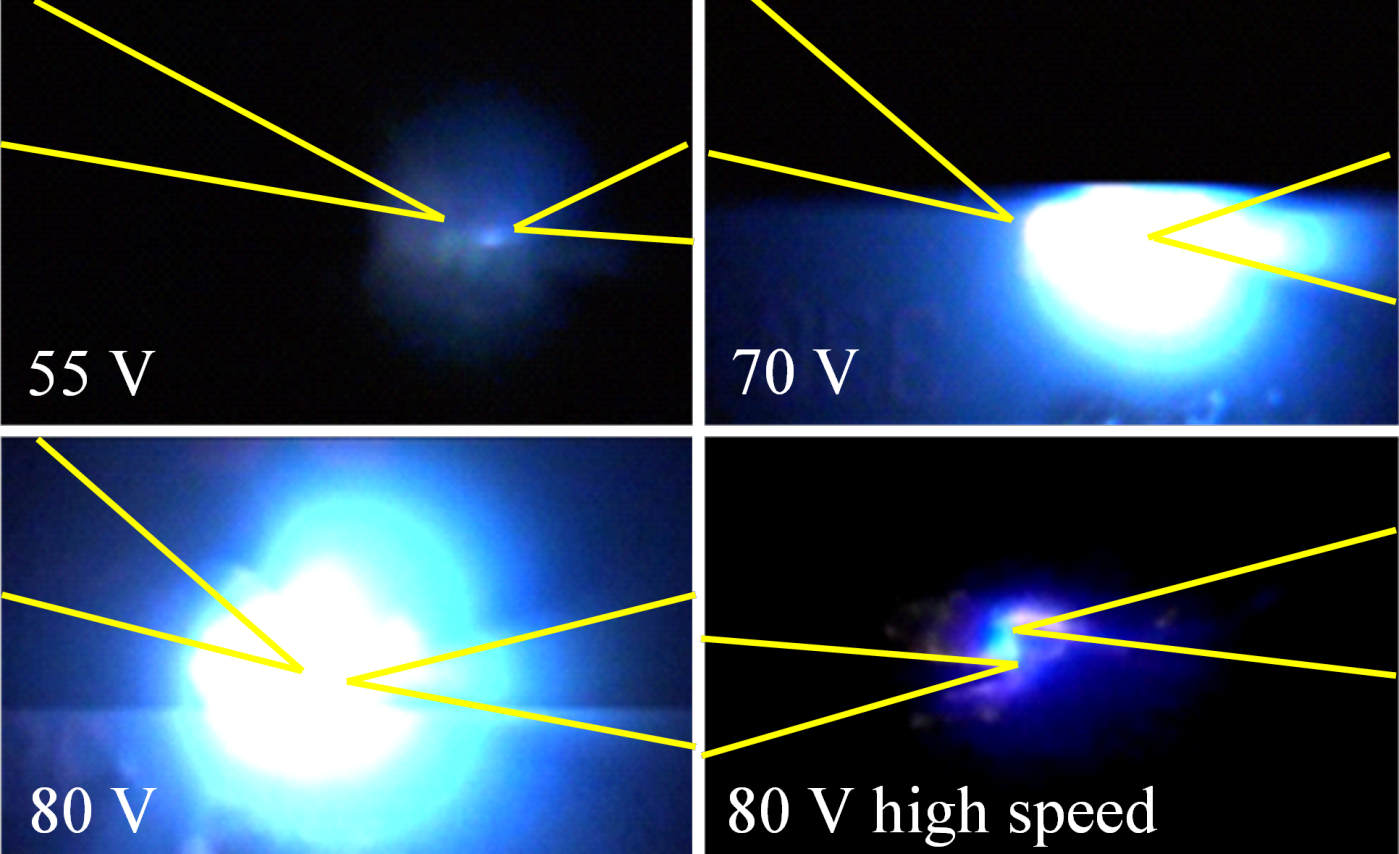
Frames extracted from high-resolution videos showing light emission from the ZnO nanorods contacted by tungsten probes during AC excitation of 10–10000 Hz with the indicated peak-to-peak voltage level. The approximate probe separation was 10–15 μm. Similar visible emissions occurred with pulsed excitation. The probe positions are as indicated in yellow.

In all experiments, bright blue and white light and high current levels were observed as the voltage level was increased (Figure 3 and Figure 4). In some cases orange–pink regions were also observed (Figure 3 and Figure 4 bottom-right). The light emission was observed to be very intense, scaling with the applied voltage and observed as multiple, brief flashes during long excitations. The emitted light seemed to follow a changing percolation path between the contacts (Figure 3, multimedia view) [20] as the materials experience changes in thermal and electrical properties over time due to the high electrical stress.

Since the ZnO nanoforest material has relatively weak mechanical properties and adhesion to the substrate, probing with tungsten tips causes some shifting of this material and so the probes are likely to touch both ZnO and the polysilicon substrate. The room-temperature electrical resistivity of the ZnO nanoforest on highly doped polysilicon is ≈6.8 Ω·cm and that of the highly doped polysilicon substrate itself is ≈0.02 Ω·cm. Therefore, the current is expected to mostly flow through the polysilicon substrate, at least in the beginning when the temperature is low. As the temperature increases, due to Joule heating, the current paths will change depending on the temperature dependence of the resistivity of these two materials. Due to the large difference in resistivity, however, it is likely that the current flows mostly through the polysilicon substrate until the electrical breakdown of air or the ZnO nanorods occurs under the high fields and lower resistivity paths open through the ZnO nanoforest.

Because of the geometrical nonuniformity of the ZnO nanoforest, the electrical resistance and electric field distribution between two probes are also nonuniform. Therefore, some segments along the current flow path are likely to undergo dielectric breakdown and joule heating earlier than others. The joule heating in turn results in sublimation of ZnO into Zn vapor and O_2_ gas and melting of the polysilicon substrate. Thus different regions between the two probes are expected to experience these thermal changes in a sequential fashion.

Typically 3.4 eV emission (UV light) is expected from ZnO if conduction band to valance band recombination is the dominant mechanism. White light can also be emitted from the ZnO structures as the excited carriers trickle down from the conduction band to valance band through multiple defect levels or because of the optical excitation of materials by the ultraviolet source [21]. Blue light has been previously observed from ZnO-based homojunction LEDs, where it is attributed to donor–acceptor pair recombination in the p-type ZnO layer [10] and also from n-ZnO/p-GaN heterojunction LEDs [22]. The superposition of electroluminescence and Fabry–Pérot oscillations has also been suggested as the possible mechanism for blue–white emission from ZnO-based homojunction LEDs [23].

**Figure 5:**
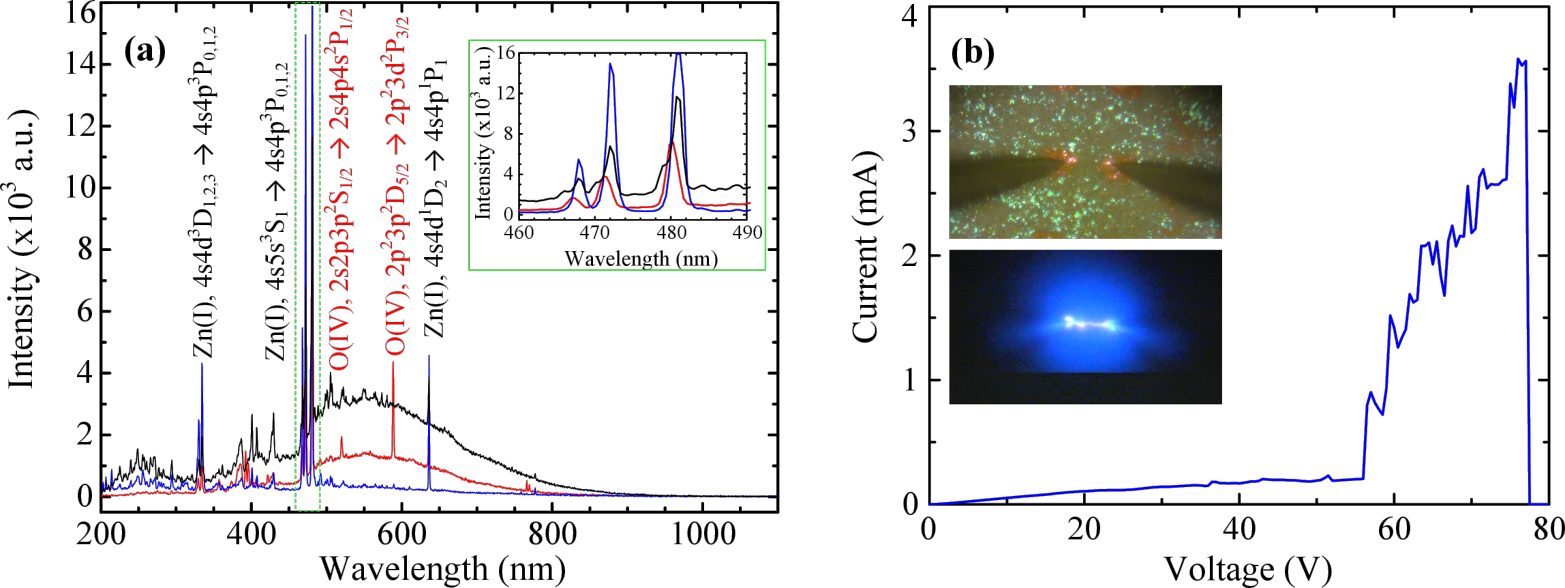
(a) Optical emission spectra obtained from DC voltage sweeping of 0–80 V (blue), AC excitation with an amplitude of 40 V at 1 kHz (red), and single pulse voltage with an amplitude of 35 V and a duration of 10 μs (black). The corresponding atomic electron transitions in the excited species are indicated adjacent to the spectral lines. The inset shows the characteristic peaks (triplet) for neutral Zn atoms. (b) Example of a DC, *I*–*V* characteristic from 0–80 V in steps of 1 V through the ZnO nanorods. The entire DC sweep was carried out in ≈10–15 s. The insets show the optical image of the probe arrangement before the test (top) and a frame from the high-resolution video of the light emission (bottom).

The spectra obtained in this work (from a large number of measurements with DC, AC and pulse analyses) consistently showed three, high intensity, fine spectral lines at approximately 481, 472 and 467 nm (Figure 5a). This triplet (in blue wavelength range in Figure 5a inset) corresponds to atomic electron transitions (AETs) in neutral zinc (Zn I) atoms. The localized orange–pink glow observed in some cases (≈635 nm) can also be ascribed to another AET of neutral zinc (Figure 5). Hence, the existence of these fine lines indicates generation of a plasma by the dielectric breakdown of air between the ZnO nanorods and of the breakdown of the ZnO structures themselves, leading to sublimation and plasma formation in the ZnO nanoforest.

**Figure 6:**
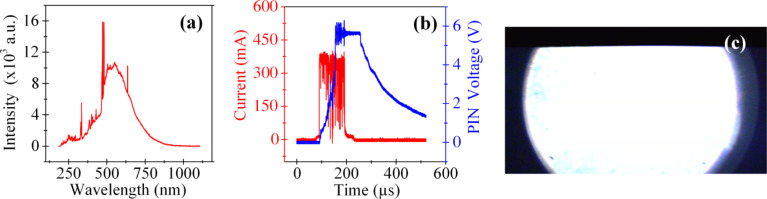
(a) Spectrum of the light emission resulting from application of a single pulse with amplitude of 45 V and duration of 100 μs. The spectral line around 480 nm was clipped due to the saturation of the spectrum analyzer. The white light (broad spectrum) content was very strong for this pulse. (b) Current and PIN output voltage versus time measured by an oscilloscope. The photodiode used for this trial was very sensitive but not fast enough to capture the details, showing slow rise and fall times and saturation. The delay was primarily due to the response time of the built-in amplifier in the PIN diode package. The fluctuations in the current indicate the recurrent formation and termination of conductive paths between the contacts. (c) A frame extracted from the corresponding light emission video.

The application of higher amplitude and longer duration voltage pulses resulted in a greater overall intensity of the emitted light (Figure 5, Figure 6). The broad spectrum (white light) emission (Figure 5 and Figure 6) can be ascribed to the free–free electron transitions in the plasma, the electronic excitations within solid ZnO and/or energy loss through multiple transitions with a very broad spectrum of trap levels [24]. Blackbody radiation may also contribute to the broad spectrum, white light emission but the spectral distribution is distinctly different than what is expected from blackbody radiation alone. The lack of broad spectrum emission in some of the DC experiments indicates a distinct difference in plasma conditions (Figure 5). The intensity of the emitted light was often strong enough to saturate the spectrum analyzer, PIN diode and the camera, even for a single µs-duration pulse (Figure 6a). The pulse measurements showed a nonsteady and high current level (Figure 6b).

**Figure 7:**
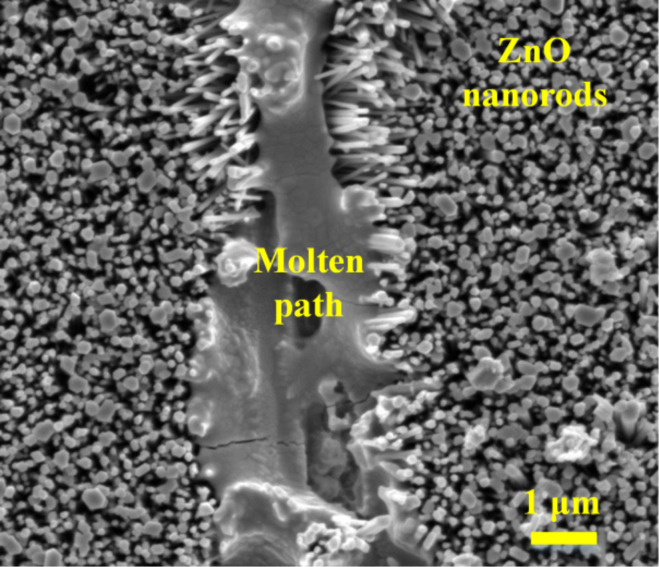
Scanning electron microscope image of the molten path due to the current flow between the contacts after a DC test showing evidence of melting and recrystallization of the material under electrical stress.

**Figure 8:**
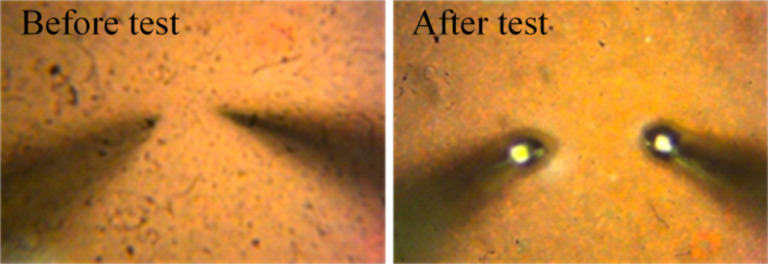
Optical microscope images showing two tungsten probes placed on a continuous ZnO nanoforest before (left) and after (right) a DC measurement test. The melting of the micrometer-sized probe tips during the electrical test suggests local temperatures above the melting point of tungsten (3422 °C).

The SEM images taken after DC electrical analysis (Figure 7) indicate melting of the materials between the contacts, and an optical image of the tungsten probes after some of the measurements (Figure 8) indicates melting of the probe tips. Hence, in some cases, the local temperature exceeds the melting point of tungsten (3422 °C), which is significantly above the sublimation temperature of ZnO (≈380 °C for the Zn surface, ≈600 °C for the O surface, and ≈1975 °C for the decomposition into zinc vapor and oxygen) [17,25].

**Figure 9:**
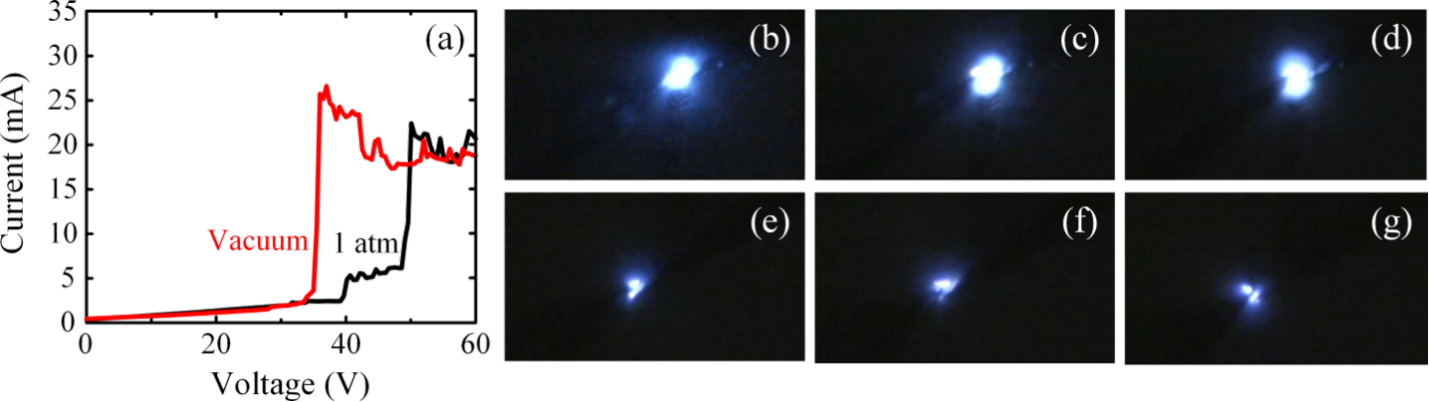
(a) *I*–*V* characteristics for two DC voltage sweep measurements from 0–60 V performed in vacuum (red) and at atmospheric pressure (black). These measurements were performed on ZnO nanorods grown on two different polysilicon microstructures of very similar dimensions. (b–g) Video frames extracted from a high-resolution camera recording for the corresponding light emission for the DC measurements at atmospheric pressure (b–d) and in vacuum (e–g).

Figure 9a shows DC voltage sweep measurements performed in vacuum and at atmospheric pressure with a probe separation of ≈10 µm on the ZnO nanorods grown on two adjacent polysilicon microstructures of very similar dimensions (length of 2.5 μm and width of 2.1 and 2.15 µm). The resulting *I*–*V* characteristics showed breakdown voltages at around 35 and 49 V at vacuum and atmospheric pressure, respectively. At lower pressure, lower temperature and hence, smaller applied electrical energy lead to sufficient vapor pressure to initiate the sublimation process [26].

The resulting light emission consisted of multiple flashes for the DC measurements both in vacuum and at atmospheric pressure. However, the light flashes in the atmospheric pressure measurements were more intense, whiter, and occurred over a broader area as compared to those in vacuum (Figure 9b–g).

The observation of light emission in vacuum implies that breakdown in ZnO, sublimation, and subsequent impact ionization are sufficient for the plasma formation process in the ZnO nanoforest, and hence, air breakdown is not required to initiate the plasma formation process.

## Conclusion

The electrical experiments performed on ZnO nanorods grown on patterned silicon microstructures lead to high intensity, blue and white light emission. The abrupt increase in current at higher fields, sharp spectral peaks and very high local temperatures (observed as significant probe melting) indicate ZnO dielectric breakdown, sublimation, and plasma generation. The sharp spectral lines correspond to atomic electron transitions of excited Zn and O species in plasma and the spectra are similar to those from previous studies on ZnO light emission through the formation of a plasma plume [27–28]. The comparison between the results obtained from vacuum and atmospheric pressure measurements also indicate a plasma process as the dominant mechanism for light emission. The confinement of the plasma within a micro-chamber to contain the evaporated material [29] together with integrated electrodes may lead to a sustainable plasma state that could be tailored for various applications.

Even though many reports on light emission from ZnO refer to solid-state electroluminescence from homojunction or heterojunction structures, our results show that plasma formation can also take place with comparable electric fields and currents and can lead to blue and white light emission.

## Supporting Information

Extracted frames from this video are shown in Figure 3.

File 1 High speed video of the light emission during DC test.
